# Recent Advances in Silver Nanostructured Substrates for Plasmonic Sensors

**DOI:** 10.3390/bios12090713

**Published:** 2022-09-02

**Authors:** Shashank K. Gahlaut, Anisha Pathak, Banshi D. Gupta

**Affiliations:** 1 Institute of Chemistry, University of Potsdam, 14476 Potsdam, Germany; 2 Department of Physics, Indian Institute of Technology Delhi, New Delhi 110016, India

**Keywords:** silver, plasmonics, sensors, surface-enhanced Raman spectroscopy, surface-enhanced fluorescence, surface-enhanced infrared spectroscopy

## Abstract

Noble metal nanostructures are known to confine photon energies to their dimensions with resonant oscillations of their conduction electrons, leading to the ultrahigh enhancement of electromagnetic fields in numerous spectroscopic methods. Of all the possible plasmonic nanomaterials, silver offers the most intriguing properties, such as best field enhancements and tunable resonances in visible-to-near infrared regions. This review highlights the recent developments in silver nanostructured substrates for plasmonic sensing with the main emphasis on surface plasmon resonance (SPR) and surface-enhanced Raman spectroscopy (SERS) over the past decade. The main focus is on the synthesis of silver nanostructured substrates via physical vapor deposition and chemical synthesis routes and their applications in each sensing regime. A comprehensive review of recent literature on various possible silver nanostructures prepared through these methodologies is discussed and critically reviewed for various planar and optical fiber-based substrates.

## 1. Introduction

The study of light–matter interaction in coinage metals has been of great interest since ancient times, and their ability to reflect light was admired for ages [1]. The other historical uses of these metals have also been widely found in artifacts such as medieval stained glasses, the Lycurgus Cup and the global antibacterial use of silver by the Greeks and Romans [2]. However, in addition to more sophisticated scientific developments and understandings in the field of the atomic world, the recent use of these metals has gained intensive interest, touching almost every possible area of science and technology. With precise control over the nano dimensions of these metals, a very strong light–matter interaction arises through the free electrons of the metals, popularly known as the field of plasmonics. Initially this light confinement was only possible with dielectrics, which is diffraction limited to areas smaller than the wavelength of light [3]. Plasmonic modes, however, can localize light in the dimensions of the supporting metallic structure, i.e., nanostructured metals can tightly concentrate and manipulate light with unrivaled accuracy in the nanometer regime. The two major roles served by metal nanostructures in plasmonics are to guide light to desired locations and to serve as nano-antennas for localized strong electric fields, thus bridging the gap between the micro and nano worlds. As a proof-of-concept, plasmons have been known for over 150 years since the documentation by Michael Faraday in 1857 [4]. However, the recent applications of plasmonics have made significant developments since the discovery of nanoscience and have opened doors for processes that were considered impossible earlier. Thus, plasmonics is considered a burgeoning field of nanotechnology with an unraveled level of control over light manipulation, with applications including catalysis [5], photovoltaics [6], superlenses [7], plasmonic circuitry [8], waveguides [9], SPASER [10], single-photon transistors [11] and sensors [3,12,13,14].

The confinement and enhancement of light by metal nanostructures have led to point-of-care plasmonic sensors achieving new competencies in their sensitivities. Plasmonic sensors are one of the first, most widely used and successful applications that have gained intensive research interest due to their ultrahigh sensitivity now reaching the single-molecule level [15]. These sensors gain an advantage from the highly enhanced electromagnetic field around the metal nanoparticles, which is extraordinarily sensitive to the surroundings. These highly sensitive plasmonic sensors can provide a sound interpretation of biological processes in a simple and noninvasive manner for an improved healthcare system and, hence, have been surveyed by recent interesting review articles [16]. Since the discovery of plasmonic biosensors about four decades ago, technological advances over time have improved the fundamental and advanced understanding of this field, while many challenges are still being actively looked into within the research community. Almost every spectroscopic technique, such as Raman [17], fluorescence [18], infrared and UV–vis [19], have gained advantages from the field of plasmonics in the context of enhanced light–matter interaction and, hence, higher sensitivity. The most important factor in the growth of this field is the explicit control of nanostructured components in terms of their ease of fabrication, cost-effectiveness and efficacy in general environments, along with unprecedented sensitivity.

Among all the metals reported for plasmonic sensors, silver and gold are considered the most useful owing to their strong plasmon resonances in the visible range, biocompatibility and stability [13]. Silver has been long known for its antibacterial applications even in historical times. It was considered an efficacious weapon against pathogens due to the interaction of silver ions with the thiol group in bacteria and proteins leading to cell death [2]. However, the efficiency of silver at nano dimensions was discovered recently due to the growing field of plasmonics. In plasmonics, the interaction of light with a metal nanoparticle is best described by Mie scattering, which provides an extinction (absorption + scattering) cross-section of a spherical nanoparticle as

(1) $$\;C_{ext}=\frac{24\;\pi^{2}R^{2}\varepsilon_{m}^{3/2}}{\lambda }\left[\frac{\varepsilon_{i}^{2}}{{\left(\varepsilon_{r}+2\varepsilon_{m}\right)}^{2}+\varepsilon_{i}^{2}}\right]$$

where R is the radius of the nanoparticle, λ is the excitation wavelength, $\varepsilon_{r}$ and $\varepsilon_{i}$ represent the real and imaginary parts of the complex dielectric function of the metal and $\varepsilon_{m}$ is the dielectric constant of the medium surrounding the metal nanoparticle [3,20]. The dielectric properties of a metal are governed by $\varepsilon_{r}$ and $\varepsilon_{i}$, which depend highly on the excitation wavelength. Thus, the interaction between a metal nanoparticle and light depends on its dielectric properties, as stated in Equation (1). Among all other factors important to the engineering of plasmonic sensors, the optical properties of the metal are key. Examining Equation (1) tells us that *C_ext_*, which signifies the strength of this electromagnetic interaction, increases to reach infinity when the denominator in the bracket approaches zero, indicating resonance in the excitation light and electronic oscillations in the metal. This will occur when $\varepsilon_{r}$ is nearly equal to −2$\varepsilon_{m}$ and $\varepsilon_{i}$ is near zero, which is not possible for dielectrics, which typically have $\varepsilon_{r}$ values greater than one. These conditions can be satisfied only by some of the metals, and their plasmon strength depends on the quality factor (QF) broadly described as the ratio of $\varepsilon_{r}$ to $\varepsilon_{i}$. The high value of QF is associated with strongly confined plasmons, whereas a low QF is associated with lossy plasmons with low C_ext_. Silver has the highest QF across most of the visible spectrum, from 300 to 1200 nm, although aluminum (Al) provides a higher value in the UV region [3,21]. Considering the interband transitions from the conduction band to higher energy levels, which weaken SPs modes, Au and copper (Cu) are limited by their localized surface plasmon resonance (LSPR) excitation, above 500 and 600 nm [22]. However, for Ag, these transitions occur far above the LSPR frequencies. The other factors to be considered for the best plasmonic material for biosensors are their toxicity and stability. Although these factors make Au the choice over silver, a passivating layer may overcome these challenges for silver very easily. The controlled synthesis techniques and overall cost of the metal will also greatly determine its feasibility for large-scale applications. Keeping in mind all the above factors, silver offers a unique choice for plasmonic biosensors owing to the strongest plasmonic resonances, cost-effectiveness and the ease of synthesis of Ag structures with controlled shape/size [3,23].

**Figure 1:**
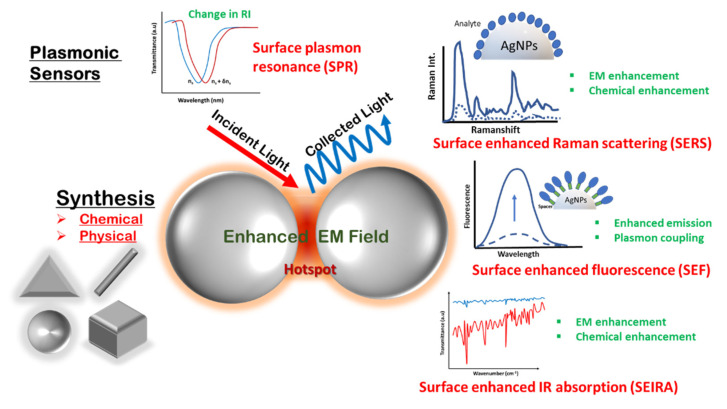
Schematic showing the scope of the review article.

This review focuses on the various synthesis methodologies for diverse silver nanostructures reported for plasmonic sensors. The aim of the article is to compile the literature specifically on silver as a plasmonic material for various sensing applications, as compared to the more generalized articles reported so far, to emphasize the peculiarity of silver over other plasmonic materials in terms of cost and efficacy [16]. It mainly focuses on the chemical methods involving solution-phase synthesis and physical methods such as vapor deposition; glancing angle deposition (GLAD); and lithographic techniques for silver nanostructures, ranging from nanoparticles, nanocubes, nanotriangles, nanorods and nanowires. Further, the review elaborates on recent spectroscopic techniques, focusing on plasmonic enhancement for biosensing methods such as SPR/LSPR, SERS, surface-enhanced fluorescence (SEF) and surface-enhanced infrared spectroscopy (SEIRS) using Ag as the plasmonic material. In addition, the focus will be on the advancements made in these fields underlying the fundamental mechanisms and applications specific to silver nanostructures for environmental and food monitoring, defense applications and biological detection. The scope of the article is schematically presented in Figure 1.

## 2. Synthesis of Silver Nanostructures

A wealth of synthesis methods has been reported and modified over time to determine a precise control over the shape and size of AgNPs. These methods may be broadly classified into two categories as physical methods (lithography, vapor deposition, microwave-assisted synthesis, laser ablation) and chemical methods (involving reduction, precipitation, photoreduction, biological/green synthesis, hydrolysis, etc.). The focus will be on the major methods reported for the synthesis of silver nanostructures for plasmonic sensors, i.e., chemical reduction, green synthesis, photoreduction, physical vapor deposition and lithography.

### 2.1. Chemical Synthesis

A fine tuning of the shape, size and composition of silver nanoparticles can greatly affect their optical, thermal, electronic and catalytic properties for a wide range of plasmonic applications. Solution-phase synthesis is the most widely accepted method for maneuvering the shape and composition of silver nanostructures with great uniformity [3]. As compared to isotropic nanoparticles such as spheres, tuning the shape of nanoparticles to obtain anisotropic structures has been a focus of researchers due to the very high enhancement of plasmonic electromagnetic fields and the possibility of tuning the resonances over a wide wavelength range, spanning from the visible to IR spectrum. Thus, the engineering of anisotropic metal nanoparticles is very important for plasmonic sensors, especially for SERS-based sensing, where tuning the size of a hotspot may lead to the detection sensitivities up to single-molecule level [24,25]. Chemical methods provide good control of the morphology of nanoparticles to obtain various shapes. Here, a metal salt (generally silver nitrate; AgNO_3_) is reduced in the presence of a stabilizer to provide innumerable possibilities of morphological control for the synthesis of silver nanostructures [14]. The reduction of Ag^+^ ions in the precursor causes elemental Ag atoms to grow into clusters and, finally, into nanostructures. The major factors controlling the growth and stabilization of nanostructures are the choice of reducing agent, stabilizer, temperature, relative concentrations and time. The reduction of metal salt/precursor may be performed in several ways, i.e., chemical reduction (using reductants such as sodium borohydride, hydroxylamine, citrate, hydrogen, hydrazine, etc.), photoreduction, electrochemical or sonochemical reduction. This reduction process is then stabilized using surfactants, ligands or organic molecules, which inhibit columbic repulsion/steric hindrance by adsorbing on the nanoparticle surface [26].

#### 2.1.1. Polyol Reduction

**Figure 2:**
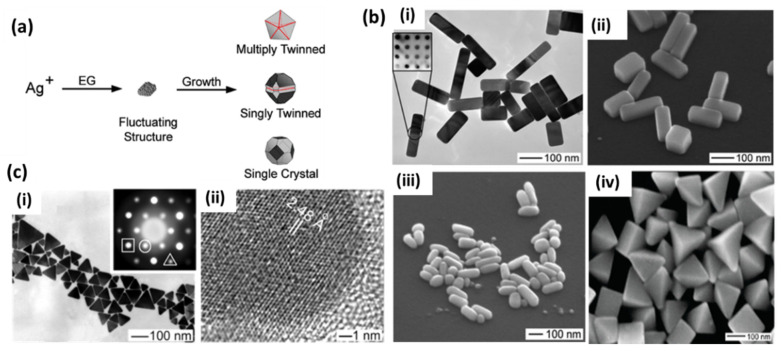
(**a**) The growth of silver nanostructures in polyol synthesis caused by the formation of silver nuclei with low surface energy twin boundary defects in order to multiply twinned, singly twinned or single-crystal seeds. The final morphology may be controlled by engineering the growth of these seeds with various parameters [27]. (**b**) (**i**,**ii**) TEM and SEM images of Ag nanobars produced with NaBr; (**iii**) single-crystal nanorice formed with the storage of nanobars in PVP; (**iv**) the formation of right bipyramids just by reducing the NaBr concentration to half, producing single-twinned seeds by causing the degree of etching to be moderated [27]. Reproduced with permission from ref. [27]. Copyright 2007, American Chemical Society. (**c**) (**i**) Triangular silver nanoplate self-assembled monolayer formed by a simple reflux of silver seeds in ambient laboratory conditions, and (**ii**) a high-resolution TEM showing single-crystallinity in the (111) direction [32]. Reproduced with permission from ref. [32]. Copyright 2003, American Chemical Society.

Polyol-based chemical reduction is the most widely accepted method for the synthesis of silver nanoparticles of various shapes and sizes. Basically, a polyol, typically ethylene glycol, is used as a solvent and reducing agent in this synthesis procedure. At an elevated temperature, silver precursor (mainly AgNO_3_) and a stabilizer or capping agent such as PVP (polyvinylpyrrolidone) are added to polyols for the reduction of Ag^+^ ions to silver nanostructures of various shapes. Several parameters, such as temperature, pH and trace ions, have been reported for the shape engineering of silver nanostructures over time to tune the SPR properties of AgNPs in polyol synthesis [27,28,29,30]. Several reports have provided detailed mechanisms for the reduction process in polyol synthesis where ethylene glycol (EG) is used both as a reducing agent and a solvent for the silver precursor. It was later proposed that EG forms glycolaldehyde in the presence of oxygen at high temperatures, and then it is the major reductant in EG-based polyol processes [31]. In these processes, the final shape of the nanostructure is controlled by the twin planes present in the seed formed at the initial stage. When silver nitrate is reduced by EG, the initial seeds may take single-twin, multiple-twin or single-crystal shapes depending on the more thermodynamically favored system, as shown in Figure 2a [27]. The nucleation and growth of one of these structures may be controlled by selecting the reaction conditions to obtain the desired shape of the final nanostructure. Single-twin seeds can be modified into right bipyramids and beams, multiple-twinned seeds can be grown into 1D nanowires and nanorods and single-crystal seeds can produce nanocubes [27]. Various morphologies have been reported by the introduction of the NaCl- and NaBr-etching of these seeds, as shown in Figure 2b. The capping agent also plays a major role in controlling the growth of selective crystal planes (100) or (111) and has been extensively used to see the effect on the morphology of the final structure [28]. On the other hand, it is believed that anisotropic structures, such as silver nanoplates, where the lateral dimension is much larger than their thickness, have the highest electromagnetic field enhancement and tunability of the LSPR peak. A simple preparation of silver nanoplates with spherical colloids of 3.5 nm was reported after refluxing in ambient light conditions. Light and the driving force provided by refluxing is assumed to transform spherical seeds into nanoplate-like structures, as shown in Figure 2c [32]. A photochemical synthesis route has also been proposed to produce silver nanoplates from citrate-capped spherical nanoparticles [33]. Here, the anisotropic growth of these structures is favored in the presence of excess Ag^+^ ions and sodium citrate. It is believed that the presence of twin planes in the seeds and the preferential capping of (111) facets by citrate are responsible for the growth of the plate structure. Later, a DFT theoretical model of this preferential binding was provided [34]. It was noticed that three-fold symmetry in citric acid matches with Ag (111). This leads to stabilization of this particular facet and growth in the lateral dimension. Zhang et al. [35] provided a detailed understanding of the formation of these kinds of structures and the most important factors involving the role of citrates and other similar carboxyl compounds. They carefully analyzed the role of the most extensively used PVP ligands and explained their trivial role in a much-simplified process of large-scale synthesis. The critical role of hydrogen peroxide (H_2_O_2_) in the formation of planar-twinned seeds to produce silver nanoplates was carefully examined and emphasized in their process. Their versatile method was later utilized by Song et al. [36] for a sensitive fiberoptic LSPR sensor.

Sun and Xia [26] reported the synthesis of silver nanocubes through such a polyol process in a very nice, shape-controlled manner. Many modifications with the involvement of Cl^−^ [37], SH^−^, HCl [38], Fe^2+^/Fe^3+^ [39], and Br^−^ [40] have been reported for monodispersed cubic particles. Most of the studies conclude that PVP chain length and the molecular weight on the final structure are important. The main hypothesis is the selective binding of PVP to (100) facets as compared to (111) facets, resulting in shapes mainly terminated by (100) facets [26].

**Figure 3:**
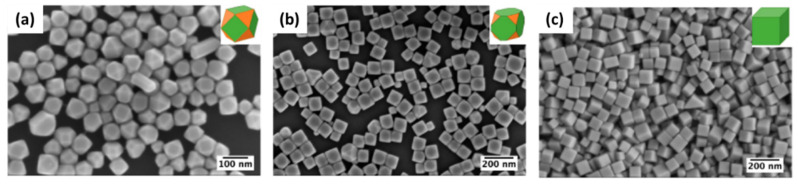
SEM images of AgNPs synthesized with varying concentrations of Cl^−^: (**a**) 3 mM HNO_3_ + 0.03 mM NaCl, (**b**) 3 mM HNO_3_ + 0.3 mM NaCl and (**c**) 3 mM HNO_3_ + 3 mM NaCl. Insets show the validation of experimental observation with abinitio thermodynamic calculations. Reproduced with permission from ref. [37]. Copyright 2019, American Chemical Society.

A very well-studied polyol synthesis was reported recently with an emphasis on the role of Cl^−^ ions in the formation of well-oriented uniform silver nanocubes [37]. Experimental and theoretical considerations were taken into account to elucidate the role of Cl^−^ in the formation of Ag nanocubes. It was found that adding HCl, on the one hand, controls the rate of AgNO_3_ reduction due to the formation of HNO_3_ and, on the other hand, controls the formation of silver cubes due to the release of Cl^−^ ions, which preferentially stabilize Ag (100). Thus, by increasing the concentration of Cl^−^, the shapes change from truncated octahedra to truncated cubes and, finally, to cubes, as shown in Figure 3.

**Figure 4:**
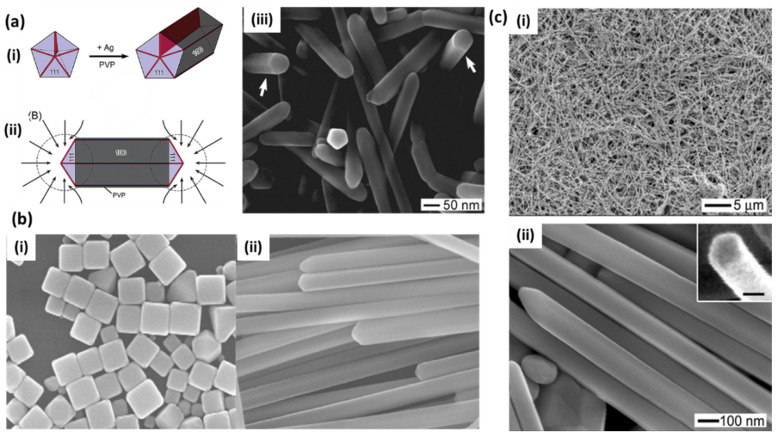
(**a**) Mechanism for the growth of pentagonal cross-section silver nanowires. (**i**) Formation of a nanorod from a multiply twinned nanoparticle (MTP) of silver due to the strong interaction between PVP and {100} facets and weak interaction with the {111} facets. (**ii**) Diffusion of silver atoms toward the two ends of a nanorod, with the side surfaces completely passivated by PVP. (**iii**) SEM image of pentagonal facet silver NWs [30]. Reproduced with permission from ref. [30]. Copyright 2003, American Chemical Society. (**b**) (**i**) Formation of nanocubes (<0.44 μM) at a low concentration of iron ions and (**ii**) nanowires at high concentrations (2.2 μM) of iron ions from silver MTPs [39]. Reproduced with permission from ref. [39]. Copyright 2005, American Chemical Society. (**c**) (**i**,**ii**) SEM images of Ag nanowires prepared with the polyol reduction of AgNO_3_ in the presence of CuCl_2_ and PVP at different magnifications [42]. Reproduced with permission from ref. [42]. Copyright 2008, Royal Society of Chemistry.

Zhu et al. [41] reported the synthesis of silver nanowires with a polyol process using PVP as the capping agent. The growth mechanism is nicely explained in terms of increasing molecular weight and the chain length of PVP due to the chemical adsorption of Ag^+^ ions on PVP chains. Tsuji et al. [29] also reported a microwave-assisted polyol process for the fast synthesis of silver nanostructures from 2D nanosheets and nanoplates at short PVP chain length (10 K) into 1D nanorods and nanowires with an increasing PVP chain length (40, 360 K). Xia’s group extensively studied the growth mechanism and many factors related to the growth of silver nanowires in the polyol process [30]. They observed pentagonal cross-section NWs in a PVP-controlled polyol process. Here, the initial molar ratio of PVP and AgNO_3_ at a particular temperature results in multiply twinned nanoparticles (MTPs) through Ostwald ripening, which further governs the growth of NWs. PVP acts as a capping and stabilizing agent that selectively passivates the {100} facets rather than {111} and facilitates the uniaxial growth of silver in 1D leading into uniform nanowires, as shown in Figure 4a [30]. Later, they also studied the role of Fe (II), Fe (III) [39] and Cu (I) and Cu (II) [42] ions in the polyol reduction process. The iron ion concentration greatly affects the nanostructure and controls the formation of nanocubes (Figure 4b(i)) or nanowires (Figure 4b(ii)) [39]. It was observed that the lower iron ion concentration results in the selective etching of MTPs, as they are unable to completely remove atomic oxygen, which is adsorbed on the surface of nanoparticles and leads to the formation of nanocubes. In a similar way, the higher concentration prevents etching and allows for the growth of twinned seeds in nanowires by removing the adsorbed oxygen. In the case of copper salt, the rapid synthesis of nanowires was attributed to the presence of both cation and anion, where Cl^−^ helps control the amount of Ag^+^ in the initial stage, and Cu(I) helps prevent oxidative etching, as shown in Figure 4c [42].

The synthesis of branched nanowires was realized by Cong et al. [43] for SERS-sensing applications using polyethylene glycol (PEG) as a solvent and reducing agent for silver nitrate and PVP as the capping agent. The branches were observed to be grown anisotropically from the defects on silver nanowire surfaces.

#### 2.1.2. Citrate Reduction

**Figure 5:**
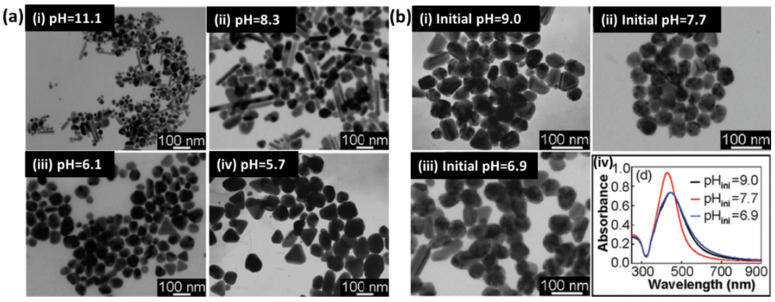
(**a**) TEM images of silver nanoparticles synthesized under pH values of (**i**) 11.1, (**ii**) 8.3, (**iii**) 6.1 and (**iv**) 5.7. (**b**) TEM images of silver nanoparticles synthesized using the stepwise method with initial pH values of (**i**) 9.0, (**ii**) 7.7 and (**iii**) 6.9, as well as (**iv**) their corresponding UV–visible absorption spectra. Reproduced with permission from ref. [48]. Copyright 2009, American Chemical Society.

The second most popular chemical synthesis technique for the production of silver nanoparticles is citrate reduction, which was proposed in 1982 [44]. Here, typically sodium citrate is used in a dual role, i.e., the reduction and stabilization of silver NPs. In a typical synthesis, an aqueous solution of sodium citrate is added to a boiling solution of silver nitrate to obtain diverse-shaped nanoparticles. A detailed study on the control of the shape and size of AgNPs in this method was conducted by Pillai and Kamat [45] using pulse radiolysis. Here, mostly large-sized silver NPs (50–100 nm) were obtained with well-defined facets. The concentration of citrate ions plays a critical role in controlling the kinetics of Ag^+^ reduction and, hence, defines the final morphology. The increase in the concentration of sodium citrate decreases the growth of silver particles by forming a complex with Ag^2+^ dimers, thereby producing larger clusters. Citrate has also been reported to be used for the photoinitiated conversion of silver nanoparticles into nanoplates [46,47]. The role of pH in this process was also investigated due to the change in the activity of citrate with pH [48]. It was found that, at high pH, citrate shows a higher reduction rate for silver precursor, resulting in spherical and rod-like nanoparticles, whereas low pH results in the formation of triangles and polygons. The TEM images of various nanoparticles prepared using this method are shown in Figure 5.

A modified citrate synthesis method has also been reported for “clean surface” nanowires without any surfactant or seed processes [49]. Here, citrate serves as a reducing agent and hydroxyl ions in NaOH facilitate the nanowires’ growth at elevated temperatures. It is believed that, although citrates provide isotropic structures at room temperature, at high temperatures, the equilibrium constant of this process may differ, and citrate binding is restricted at certain crystal faces, allowing for the growth of wire structure. The concentration of hydroxyl ions was found to be responsible for the growth of nanowires and their aspect ratios. The advantage of these methods is the clean surface, as compared to template-directed methods (using polymers and surfactants), where multiple washings are required to remove the template for sensing applications such as SERS. Molecular dynamics simulations were also provided by the same group to better understand the growth of these nanowires in citrate-mediated processes [50]. The citrate-capped AgNPs have been the choice for SERS-based studies for a long time. The LSPR properties of single and dimeric silver nanoparticles prepared with citrate reduction have been studied thoroughly with electron microscopy [51] and later applied to single-molecule SERS studies [52].

More recently, the field of the green synthesis of silver nanostructures has picked up pace due to its ample availability and environmentally friendly route [53,54,55,56]. A green synthesis approach has been proposed for spherical and crystalline silver NPs, self-assembled on NH_2_-modified glass substrates using citrus peel extracts from orange fruit (AgNP-Ora), tangerine fruit (tangerine fruit, AgNP-Tan) and lemon fruit (AgNP-Lem) [57]. These substrates were utilized for SERS studies of 4-aminobenzenethiol, rhodamine 6G and methylene blue as Raman probe molecules in µM concentrations. However, a lack of shape-controlled structures still limits their use in real applications and provides a lot of room for the research community. Various other methodologies such as homogenous and heterogenous seed-mediated growth [58,59,60], template-directed growth [61,62,63,64] and light-mediated [65] chemical synthesis methods have also been reported for AgNPs.

Therefore, till now we discussed major routes of chemical synthesis of AgNPs. However, a major issue with the colloidal substrate is the tendency to aggregate after the addition of analyte solution, which makes the colloid unstable and often leads to the poor reproducibility of the signal. In addition, there is very low control over the tuning of analyte-NP surfaces in colloidal nanoparticles, leading to the comparatively low enhancement of spectroscopic signals. Moreover, the transfer or deposition of colloidal NPs on any surface has always been a concerning factor in many applications due to inherited inhomogeneity at the macro- and nano-level [66,67]. For example, deposition on curved substrates, e.g., optical fiber, is not an easy task. Binding chemistry to attach NPs needs to be meticulously designed to ensure uniform coating. Hence, although the wet synthesis of NPs is found to be a very easy and cost-effective synthesis method, at the same time, the response and enhancement are compromised. This leads to very few commercially available plasmonic sensors fabricated with colloidal nanoparticles due to difficulty in reusability, homogeneity and disposal after every use. In contrast, physical vapor deposition techniques, such as thermal and e-beam evaporation, sputtering, etc., ensure very high reproducibility, purity, uniformity and the high enhancement of plasmonic signals. These substrates may prove to be very reliable plasmonic nanosensors with great stability for commercial and large-scale fabrication needs. Thus, the next section is devoted to discussing the physical deposition techniques for plasmonic sensors.

### 2.2. Lithography-Based Silver Substrates

Photolithography is the most used technique for the deposition of patterned arrays of metal and semiconductor materials on planar substrates, especially for device fabrication. Due to the diffraction limit of light, it does not fulfill the resolution requirement for smaller feature sizes. Electron beam lithography (EBL) and focused ion beam lithography (FIB) are top-down approaches utilizing polymeric resists to fabricate masks for the deposition or etching of metal with high resolutions. They have the ability to control the size, shape and periodicity of the nanostructured substrates [68]. In EBL, a focused electron beam is scanned over a substrate, mainly silicon (Si), through a programmed desired mask. The Si wafer is already spin-coated with a special polymer layer called a resist. This polymer is sensitive to exposure to e-beams. It could be a positive or a negative resist. In the case of a positive resist, for example, polymethyl methacrylate (PMMA), the exposed area breaks down after being illuminated by an e-beam, and then it dissolves into a solvent (developer). The substrate is coated with a silver layer of desired thickness using a metal evaporation process, and the rest of the metal, which is a resist, is lifted off, leaving a regular array of silver nanoislands. The resolution capability of EBL has repeatedly been reported to be up to 10 nm [68]. Similarly, in FIB, a gallium ion (Ga^+^) beam is used in place of an electron beam for metal deposition and etching. However, FIB includes the possibility of maskless deposition. Very thin, rectangular nanoarrays and overhanging nanostructures with very high resolution are possible with FIB [69,70]. The major issue with these conventional lithographic techniques is that they are expensive, time-consuming and need sophisticated equipment, which limits their use for scalable fabrication. Consequently, a facile lithographic technique known as nanosphere lithography (NSL) was developed by Fischer and Zingsheim [71]. NSL is cost-effective and timesaving as compared to the aforementioned techniques. In parallel, Deckman and Dunsmuir also successfully illustrated this technique in 1982 by preparing a monolayer of spherical particles on a substrate to use as a mask for the fabrication of nanomaterials and called this technique “Natural Lithography” [72].

**Figure 6:**
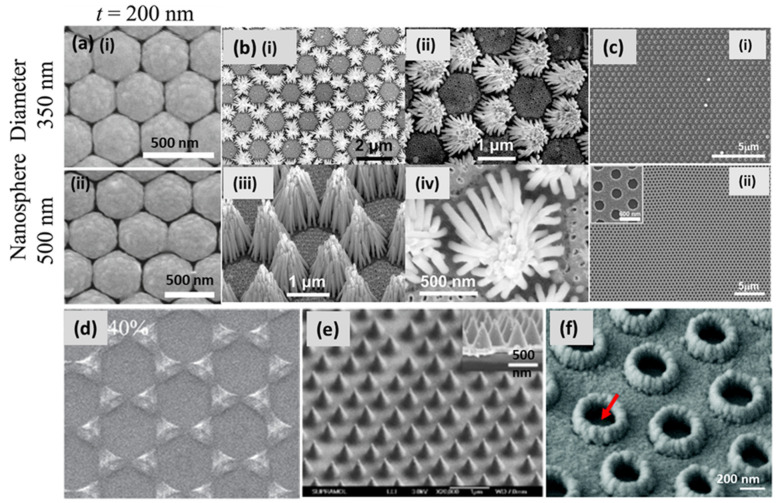
Ag nanostructured substrates fabricated by NSL. (**a**) Top-view SEM images of a 200 nm thick layer of Ag, deposited on monolayers of polystyrene nanospheres of (**i**) 350 nm (**ii**) 500 nm diameter at a tilt angle of θ = 55° [74]. Reproduced with permission from [74]. Copyright 2020, IOP. (**b**) SEM images of as-prepared Ag-nanorod bundle arrays: (**i**,**ii**) top views at different magnifications, (**iii**) side view of the bundle arrays and (**iv**) top view of a single bundle of Ag-nanorods [75]. Reproduced with permission from [75]. Copyright 2016, John Wiley & Sons. (**c**) (**i**) SEM showing an Ag nano-mesh film with a PS 600 nm nanosphere template after 50 nm thick Ag deposition and (**ii**) the resulting large area (~30 × 30 μm^2^), a single-crystalline, hexagonal-aligned hole array [76]. Reproduced with permission from [76]. Copyright 2009, American Chemical Society. (**d**) Ag–Cu mixed phase nanopatterns at the calculated composition of Ag 40% using shadow nanosphere lithography and glancing angle co-deposition [77]. Reproduced with permission from [77]. Copyright 2017, IOP. (**e**) SEM image (45° tilting views) of hollow nanocones with a height/diameter of 500/350 nm; the insets show the cross-sectional view of the corresponding samples [79]. Reproduced with permission from [79]. Copyright 2014, American Chemical Society. (**f**) SEM of disk-in-volcano arrays at a 30° tilting view with a bottom hole diameter of D = 340 nm; the top-hole diameter is d = 220 nm; the height of the volcano is H = 250 nm; the height of the disk is h = 100 nm; and the total thickness of the film = 150 nm. The red arrow points to the disks inside the volcanos [80]. Reproduced with permission from [80]. Copyright 2015, Royal Society of Chemistry.

NSL, also known as natural lithography (as previously noted), colloidal lithography and shadow nanosphere lithography, is a promising technique for the fabrication of two-dimensionally arranged periodic, disconnected nanostructures on both rigid and flexible substrates. In this technique, a monolayer of colloidal nanospheres is deposited on the substrate, e.g., Si or glass, to use the pattern for the mask [72,73]. Over time, different methods have been invented, e.g., a self-assembled monolayer of polystyrene nanospheres on the air/water interface, spin-coating, rubbing, etc., in order to design a template for fabrication in a large area. Creating a closely packed hexagonal monolayer of spherical particles on a larger area of the substrate, with a high-quality, single-crystalline domain, is still a challenging task. Recently, Zhao’s group extensively exploited NSL for patterning 2D arrangements of tunable plasmonic substrates, also shown in Figure 6a [74]. Zhu et al. demonstrated Ag nanorod bundles array vertically when grown on a Cu/Au substrate using colloidal lithography combined with the binary-template-assisted electrodeposition method, as shown in Figure 6b [75]. Lee et al. demonstrated a defect-free silver nanohole (diameter 300 nm) array over a millimeter-sized area. A colloidal, self-assembled monolayer of polystyrene nanospheres was deposited on a substrate at the air–water interface. Then, reactive ion etching (RIE) was employed to shrink the nanospheres before silver deposition, which led to the formation of a metallic nanohole array (shown in Figure 6c) after nanosphere removal with extraordinary optical transmission [76]. The fabricated array exhibited a high density of hotspots for the SERS-based sensing of phenolic pollutants. Ingram et al. demonstrated Ag–Cu mixed phase nanopatterns at different compositions of metals, combining shadow nanosphere lithography and glancing angle co-deposition (Figure 6d) [77]. Combining NSL with oblique angle deposition, they designed a transparent metallic nanohole array over a large surface area with improved electrical properties [78]. A thick layer of Ag (>100 nm) was made transparent with a periodic array of hollow nanocones using NSL. The optical transmission was measured with the height of the tips and the diameter of the holes. A SEM image of hollow nanocones, with a height/diameter of 500/350 nm, is shown in Figure 6e [79]. By extension, the same group demonstrated a surface plasmon sensor by fabricating disk-in-volcano array structures using NSL, as shown in Figure 6f [80].

In another report, a close-packed monolayered polystyrene (PS) sphere array was successfully prepared using the H_2_O_2_-assisted air–water interfacial floating method. The advantage of H_2_O_2_ over water is the decreasing number of second-layer defects and the promotion of the self-assembly of PS nanospheres to form a close-packed SAM [81]. For more details about NSL, readers are referred to a comprehensive review [82] and other useful research articles [83,84].

NSL, nonetheless, has been found to be a promising and easy-to-use technique to fabricate a regular pattern of plasmonic nanostructures in a 2D arrangement, but, like other techniques, it also has some limitations, for example, the moderate ability to tune the shape of nanostructures and fabrication on large surface areas. In addition, self-assembled metallic colloid is another method to produce regular-patterned nanostructures [85]. Yet again, the reduction of metallic salt takes place on the substrate, which can lead to random aggregation and low reproducibility. To overcome all these challenges, the entire thin film community came up with a solution by using physical vapor deposition (PVD) techniques.

### 2.3. Glancing Angle Deposition (GLAD)

In general, PVD comprising thermal evaporation, e-beam evaporation and sputtering has been extensively used for uniform thin film deposition on a large-area solid substrate. If the vapor flux is incident on the substrate at a normal incidence angle, a thin film or nano-island growth be achieved [86]. Conversely, if the substrate is placed at an angle (generally > 70°) from the incoming vapor flux, a new era of sculptured thin film deposition starts. This specialized PVD method is generally known as oblique angle deposition (OAD) or glancing angle deposition (GLAD) [87,88,89,90]. As this tool has much potential to fabricate a variety of nanostructured substrates with high purity, uniformity and reproducibility, it has been accepted by numerous researchers worldwide, specifically those working on plasmonic sensors [89,91]. GLAD is a specialized version of physical vapor deposition (PVD), offering a provision of manipulation of the substrate orientation during thin film deposition in a high-vacuum chamber. The substrate can be rotated in polar and azimuthal directions with respect to the direction of the incoming vapor flux. The ballistic shadowing effect plays a major role in the foundation of columnar thin films in GLAD, which is only possible when the incoming vapor flux is well collimated toward the substrate, which could be glass, Si or sapphire. A large angular spread in incoming vapor flux may result in poor shadowing [88]. There are two prominent approaches to obtaining collimated vapor flux: a large distance between the vapor source and substrate or physical obstacles that select a subset of the uncollimated vapor flux. A great distance from the source to the substrate not only improves the collimation of the incidental vapor flux, but also increases the number of collisions of atoms before reaching the substrate. Vapor’s mean free path is another important parameter that should be larger than the source–substrate distance in GLAD configuration. One needs to optimize all the deciding parameters to attain high-quality nanostructured films using GLAD. To evaporate the metal powder or pellets, electron beam and thermal sources are usually employed in GLAD depositions. At a larger angle of incidence (>75°), the low surface diffusion of adatoms and the shadow cast by the larger nucleation collectively result in one-dimensional columnar growth. Initially, the incident atoms are randomly deposited on the substrate, having some defects or roughness. Following the Volmer–Weber growth model, these adatoms form small islands and then 3D nuclei on the substrate. As deposition proceeds, the initial nucleated islands start projecting ballistic shadows on the surrounding region. The larger islands receive more than the smaller ones or the shadowing regions, which converts them into a columnar structure tilted in the direction of the incident flux. The direct and spontaneous growth of nanostructured thin films of a wide range of materials on various substrates can be easily attained using GLAD [87,88,89,90,91]. M.J. Brett et al. [87,88] pioneered this technique, and then it was widely explored by many researchers for the fabrication of various metals and insulator-sculptured thin films.

**Figure 7:**
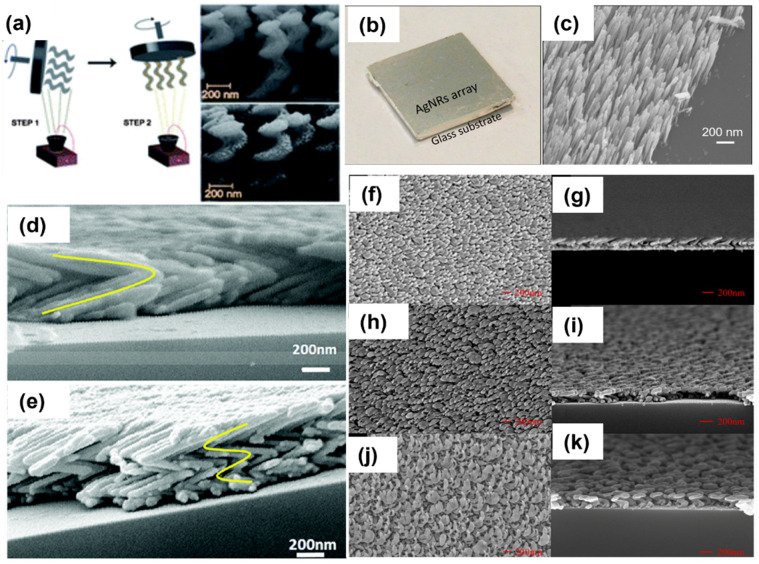
(**a**) Schematic of the GLAD technique used to fabricate a dielectric 3D template in the first step, followed by the evaporation of Ag metallic islands in the second step. SEM images of the nanostructured surfaces without (top) and with (bottom) the Ag metallic islands [92]. Reproduced with permission from [92]. Copyright 2013, Royal Society of Chemistry. (**b**) Photograph of the substrate, (**c**) SEM image of Ag nanorod array fabricated on glass substrate by using GLAD [93]. Reproduced with permission from [93]. Copyright 2017, American Chemical Society (**d**) SEM images of zigzag silver nanostructures on Si substrates with different bending numbers: two arms; (**e**) four arms. Yellow lines indicate the approximate arm positions [96]. Reproduced with permission from [96]. Copyright 2017, Royal Society of Chemistry. Top-view and cross-section SEM images of Ag nanohelix arrays deposited at different deposition angles: (**f**,**g**) 80°, (**h**,**i**) 83° and (**j**,**k**) 86° [97]. Reproduced with permission from [97]. Copyright 2017, MDPI.

In recent years, a large number of 3D nanostructures, e.g., columns, rods, helices, zigzags, springs, etc., have been obtained using GLAD, as shown in Figure 7. In the scope of this article, we focused on various silver-sculptured thin films manifested by GLAD. Ag-decorated SiO_2_ helical films were deposited on large-area transparent substrates, and the plasmonic chiroptical properties of these arrays were studied using circular dichroism. The chiral nanohelices are shown in Figure 7a [92]. This could be an exceptionally facile method to fabricate metamaterials on any solid surface. In recent years, GLAD-fabricated nanocolumnar pure Ag nanorod arrays were used for gas-sensing applications for the first time (Figure 7b) [93]. A drastic change in the colorimetric and water wettability properties of AgNR arrays was observed in the presence of a low amount of H_2_S gas. The same feature of these substrates was exploited for the detection of viability and antimicrobial resistance in bacteria [94]. Further, sulfurized AgNRs were utilized in the form of multifunctional material Ag-Ag_2_S nanoheterostructures, on which photocatalysis, hydrogen evolution, SERS and antimicrobial properties were investigated [95]. In another report, the same group studied the effect of a number of Ag arms in a zigzag array (shown in Figure 7d,e) on the EM enhancement of the SERS signal [96]. Moreover, Jen et al. fabricated a chiral Ag nanohelix array, and the effect of GLAD parameters (angles, deposition rate and rotation speed) on their growth was studied in detail (Figure 7f–k) [97].

The fabrication of silver film on patterned or templated substrates can also be realized using GLAD. Aligned tilted AgNR arrays have been fabricated on commercial compact discs (CDs) and digital versatile discs (DVDs) by using this technique. Before depositing Ag, the thin polycarbonate protective coating found on these discs was removed by immersing them in a concentrated nitric acid solution for a certain amount of time. The disc was then washed and rinsed with deionized water and blow-dried with nitrogen gas. It was then loaded into a vacuum chamber for metal evaporation at an angle of 86° with respect to the vapor direction. Due to the shadowing effect caused by deposition, the resulting hybrid structure acts as an effective anisotropic grating, with a period for the disc. The structure exhibited an optical anisotropy that depends on the polarization of the incident light. The variations in the plasmonic resonant shift with respect to the aspect ratio of the AgNRs and film thickness were studied [98,99]. Ag nanostructured grating substrates show significant plasmonic enhancement and, therefore, have been exploited for surface plasmon-based refractive index sensors [100,101] and SERS-based sensors [102,103].

There are tremendous possibilities in designing and fabricating numerous nanostructures by controlling deposition parameters, such as incident angle *θ*, azimuthal angle *φ*, substrate rotation, deposition rate, substrate temperature, material to be deposited and substrate [104,105]. Sculptured thin films fabricated using GLAD have potential applications in numerous fields, and above all, plasmonics is the major one. The GLAD-fabricated substrates emerged to overcome the challenges of uniformity and reproducibility in SERS substrates, which were extensively reviewed recently [106]. The simplicity, flexibility, cost-effectiveness and versatility to deposit a variety of materials are the main features that make GLAD stand out as the most promising tool for micro- and nanofabrication [107].

## 3. Detection Methodologies for Silver Nanostructure-Based Plasmonic Sensors

The field of sensing has found its quintessential implications in next-generation devices for environmental monitoring, food safety, defense applications, medical diagnostics and the development of smart medicines, etc. Portable, fast and easy-to-use sensors are in demand with the fast pace of development in every area of human life. Plasmonic sensing has continuously emerged as a vital tool for fast and specific detection in the past decade. The enhanced and tightly confined electromagnetic (EM) field associated with plasmonic nanostructures influences almost all optical spectroscopic methods and is at the forefront of rapidly emerging surface-enhanced spectroscopies such as SERS, SEF and SEIS. In the next sections, the fundamentals of these technologies and their applications in sensing will be discussed in the context of silver nanostructures.

### 3.1. Surface Plasmon Resonance

Over the past two decades, the field of plasmonic sensors based on SPR and LSPR schemes has sufficiently matured, with a plethora of label-free and continuous monitoring applications. Since the initial reports studying interactions on metal surfaces and gas-sensing, there has been a rapid development in the fundamental mechanisms and applications of this field, making it a prime tool to monitor label-free surface interactions in real-time. Recent developments have focused on sensitivity enhancement and the quantum aspects of these sensors, as well as their applicability in the form of industrial devices that will clearly revolutionize the field of optical sensors [108,109]. The goal now is to focus the attention of the SPR community on advancing the technology from proof-of-concept to industrial devices, such as the first one commercialized in 1990 by Biacore, by taking note of thousands of articles focusing on the quantification of clinical and environmentally relevant analytes at desired concentration levels. The fundamentals of SPR sensors have been extensively reported in various reviews and books over the past decade, with various possible transducer geometries such as the prism-based Kretschmann configuration, planar waveguides, gratings, optical fibers and many more [110,111,112,113,114,115,116,117]. A brief overview of the concept is provided here for the purpose of completion.

Plasmons are the collective electron oscillations in metals that can be confined to metal–dielectric interfaces known as propagating surface plasmons (PSPs) or to the surface of a metallic nanostructure (of dimensions less than the wavelength of light) that are termed localized surface plasmons (LSPs). The coupling of these modes to incident light gives rise to resonances that strongly depend on the compositions, shapes and sizes of the metal nanostructure, as well as the dielectric properties of the surrounding medium, making it a critical tool to probe surface processes. The EM field associated with SPs and LSPs is bound to the surface and decays exponentially in the surrounding medium with decay lengths of ~30 nm and ~ 200 nm, respectively. Thus, the sensors based on these mechanisms are extremely sensitive to changes near the surface. SPR and LSPR sensors are based on modulation in the refractive index of the sensing layer around the metallic nanostructure due to physicochemical interaction with the analyte.

**Figure 8:**
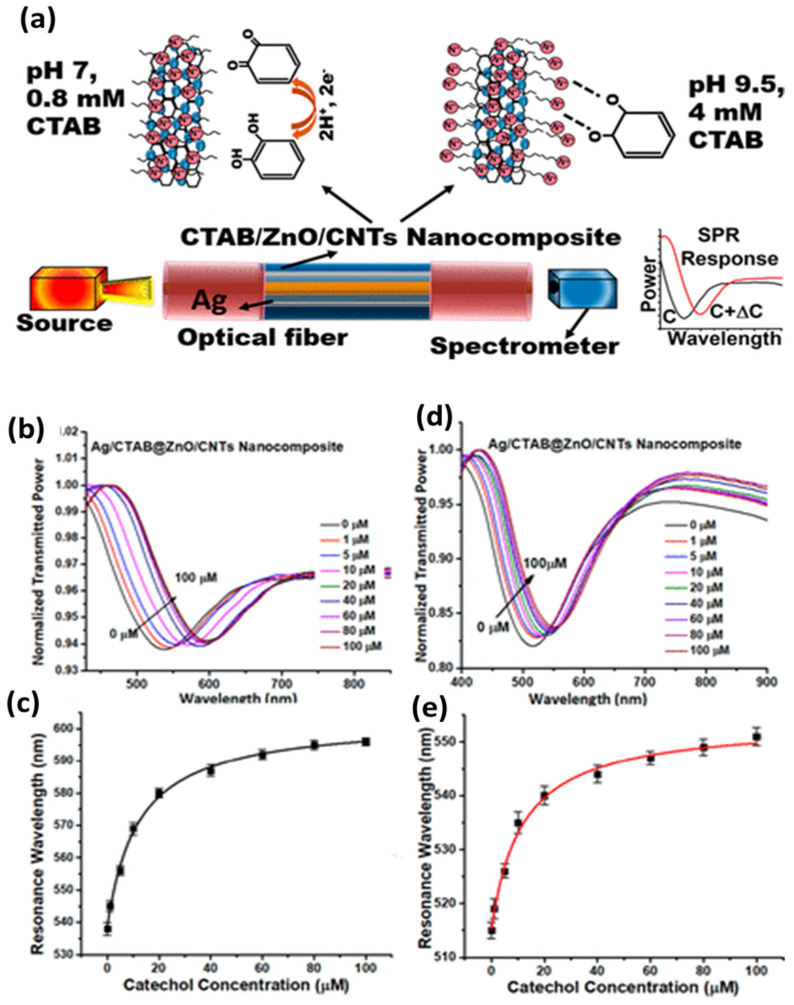
(**a**) The sensing mechanism and experimental setup for the sensing of catechol using silver thin film as the plasmonic material and CTAB-functionalized ZnO/CNT nanocomposite as the sensing layer. (**b**) SPR response and (**c**) calibration curve for the regime: pH of the sample, 9.5, and CTAB concentration = 4 mM. (**d**) SPR curves. (**e**) Calibration curve for the regime: pH of the sample, 7.0, and CTAB concentration = 0.8 mM. Reproduced with permission from [118]. Copyright 2020, American Chemical Society.

The sensors based on silver nanostructures are discussed here with widespread applications in environmental monitoring, biosensing, food safety and defense security. Recently, a fiberoptic SPR sensor based on silver thin film for the sensing of the phenolic compound catechol, a dangerous environmental pollutant, was reported [118]. The thermal deposition of 40 nm silver film was used as a plasmonic metal with the sensing layer comprising a ZnO/CNT nanocomposite for the interaction of catechol. The nanocomposite was functionalized with cetyltrimethylammonium bromide (CTAB), which governs the sensing performance of two pH regimes. The schematic of the sensing mechanism, the experimental setup and the SPR response of the two pH regimes are shown in Figure 8. The performance was analyzed over a concentration range of 0–100 µM, with the detection limit reaching 0.1 µM, making it feasible for real applications.

Moreover, Ag has also been incorporated with other metals and semiconductors to enhance their optical and electrical properties in low-dimensional hybrid nanomaterials. Nonlinear optical absorption and electrical conduction in multiwalled carbon nanotubes (MWCNTs) were observed to be enhanced when they are coated with AgNPs. The optical nonlinearities exhibited by Ag/MWCNTs can be attributed to the saturable absorption regarding the absorption band associated with the SPR of AgNPs [119]. In another report, MWCNTs decorated with AgNPs were demonstrated to improve acetone gas-sensing at room temperature using the resistive sensing method [120]. Some other SPR-based sensors utilizing carbon nanomaterials and silver-based nanocomposites were reviewed by Gupta et al. [121]. In addition, Ag-metal oxide nanohybrid systems have been widely explored in sensing applications [86,122].

Recently, a self-referenced optical fiber LSPR sensor for the detection of environmental pollutants, mercury and hydrogen peroxide (Hg^2+^ and H_2_O_2_) was reported, using the layer-by-layer nanoassembly of silver and gold NPs [123]. The plasmonic substrates were prepared using the electrostatic attraction between poly(acrylic acid) (PAA)-capped AuNPs and poly(allylamine hydrochloride) (PAH)-capped AgNPs. The AuNPs showed a higher affinity for Hg^2+^ as compared to other metal ions and resulted in a wavelength shift of LSPR spectra, whereas the AgNPs showed degradation due to the catalytic decomposition of H_2_O_2_. Thus, a self-referenced sensor was realized as SPR wavelengths of either Ag or Au shifted upon exposure to Hg^2+^ and H_2_O_2_. Similarly, numerous sensors have been realized based on plasmonic silver for the detection of environmental analytes such as nitrates, arsenic ions [124], phenolic compounds [125], hydrogen peroxide [126], methane gas [127] and ammonia gas [86].

**Figure 9:**
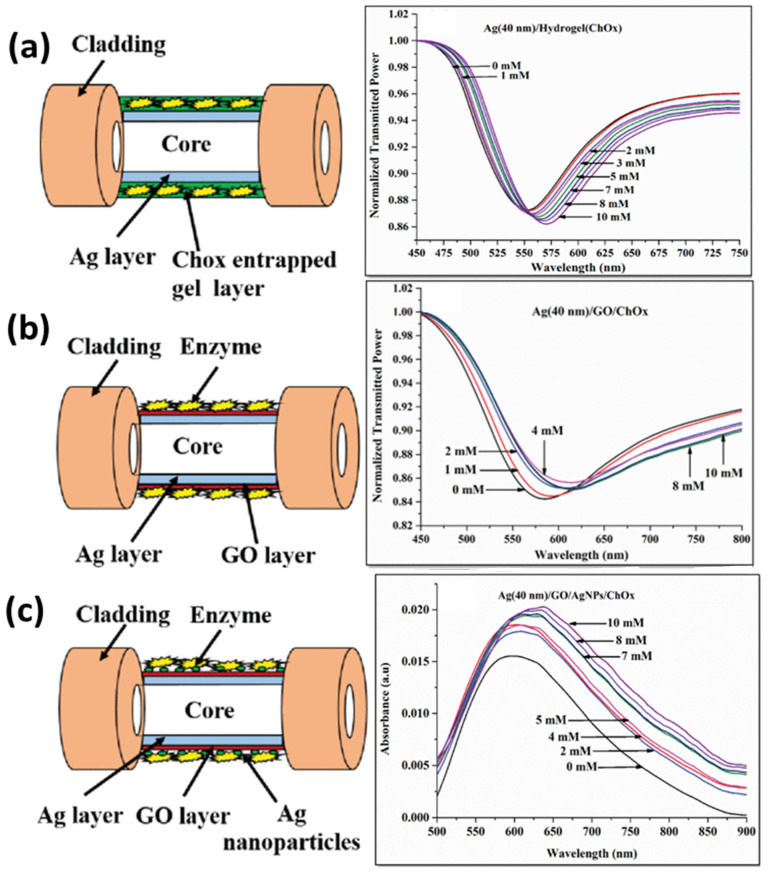
Schematic and corresponding SPR response for (**a**) ChOx-entrapped hydrogel layer over Ag thin film, (**b**) GO nanosheets along with ChOx over Ag thin film and (**c**) ChOx immobilized on AgNPs embedded in PVA over a GO nanosheet probe. Reproduced with permission from [128]. Copyright 2018, IEEE.

A great deal of progress has also been made in the field of SPR-based biosensors using silver nanostructures. A silver nanoparticle-based SPR/LSPR fiber probe was reported for the sensing of cholesterol using the enzyme cholesterol oxidase (ChOx) and graphene oxide (GO) sheets in the sensing layer [128]. The performance of three types of probes shown in Figure 9 was compared and optimized for a range of pH. The first probe involves a ChOx-entrapped hydrogel layer over Ag thin film; the second probe has a layer of GO nanosheets, along with ChOx, over Ag thin film; and the third probe contains ChOx immobilized on AgNPs embedded in PVA over GO nanosheets. It was noticed that the probe with silver nanoparticles and GO showed the best response due to the combined effect of SPR and LSPR, along with the role of hydrogen peroxide decomposition due to AgNPs. The third probe showed the best sensitivity and the lowest limit of detection (LOD) value for cholesterol, reaching 1.131 mM.

**Figure 10:**
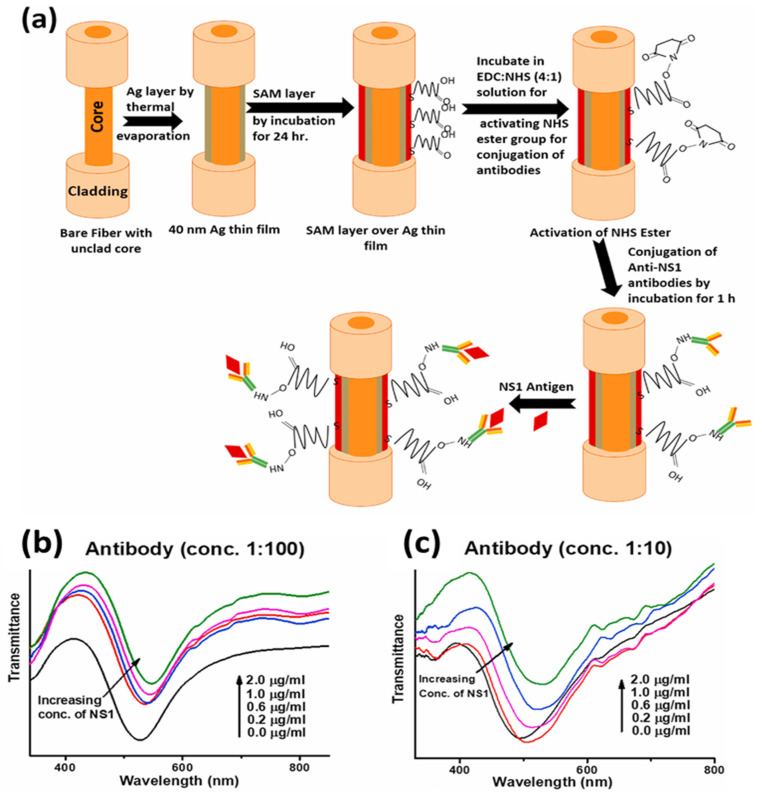
(**a**) Fabrication steps of the fiber probe for NS1 antigen detection, and (**b**,**c**) SPR response curves with changes in NS1 concentration at two antibody concentrations. Reproduced with permission from [129]. Copyright 2022, Elsevier.

Similarly, very recently, a biosensor for detecting NS1 antigen related to dengue fever that uses silver thin film and an antibody–antigen interaction mechanism was reported [129]. The probe was optimized for various self-assembled monolayers of alkanethiols for effective antibody attachment, which finally governs the stable interaction with the NS1 antigen. The schematic for the probe design and interaction mechanism is shown in Figure 10a. The SPR response is depicted as a red shift in the resonance wavelength with an increasing antigen concentration (Figure 10b). The feasibility of the sensor was tested for dengue-positive blood serum samples, underlying its usage in direct clinical applications for early dengue diagnosis with a very fast response time of 20 min.

A similar rapid biosensor was also reported for SARS-CoV-2 detection using a silver nanotriangle array as an LSPR sensing platform functionalized with human angiotensin-converting enzyme 2 protein (ACE2). The sensor selectively and rapidly detected the COVID virus, with LOD values of 0.83 pM, 391 PFU/mL and 625 PFU/mL in the spike RBD protein and CoV NL63 in the buffer, and untreated saliva providing a very effective alternative to rapid antigen tests [130]. A plethora of biosensor reports are available in the literature for the silver-based LSPR sensing of various biomarkers and bioanalytes for, e.g., glucose [131], ErbB2 breast cancer biomarker [132], triacylglycerides [133], cysteine [134], endotoxins [135] dopamine [136], ascorbic acid [137], urea and glucose [138].

**Figure 11:**
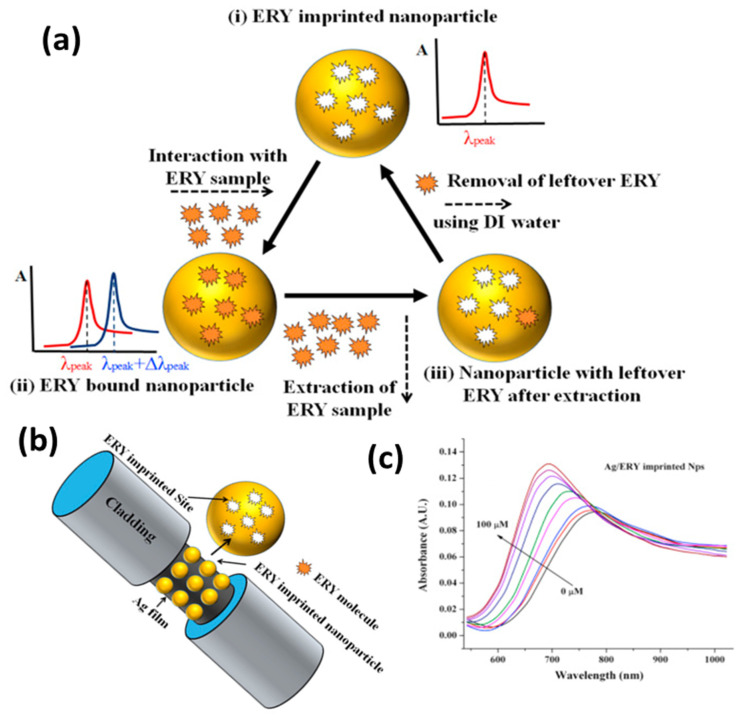
Application of silver-based SPR sensors for food safety. (**a**) Sensing mechanism in a MIP-based approach for the sensing of ERY in milk and honey. (**b**) Optical fiber probe. (**c**) SPR response with an increasing concentration of ERY. Reproduced with permission from [140]. Copyright 2017, Elsevier.

Another important domain of SPR-based sensors is food monitoring and homeland security. A recent review summarizes plasmonic sensors for food security [139]. Shrivastav et al. proposed a molecular imprinting (MIP)-based fiberoptic SPR sensor for the detection of erythromycin (ERY) residue in milk and honey [140]. To overcome the limitation of the lower sensitivity of bulk MIP, MIP nanoparticles prepared with the two-phase mini-emulsion method were utilized. The sensing principle relies on a change in the refractive index of the MIP layer coated over the silver thin film on the optical fiber. ERY is imprinted in the MIP matrix during the synthesis step using certain monomers and a cross-linker. Thus, during the sensing step, ERY molecules bind noncovalently with these imprinted sites, bringing the change in the refractive index with a changing concentration. A schematic of the sensing mechanism, optical fiber probe and SPR response for ERY quantification are shown in Figure 11. ERY was spiked in milk and honey to test the feasibility of the probe in a real sample analysis. Another report also explored the sensing of the adulterated, nitrogen-rich compound melamine based on a similar MIP technique [141] for food safety.

**Figure 12:**
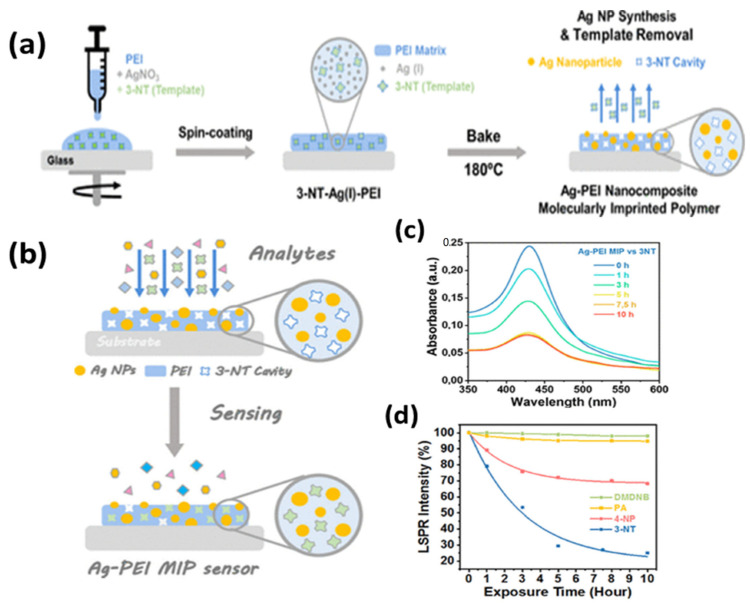
(**a**) Synthesis of a MIP layer for detecting explosive taggant 3-NT; (**b**) sensing mechanism; (**c**) SPR response; and (**d**) selectivity. Reproduced with permission from [142]. Copyright 2021, American Chemical Society.

A very recent report on homeland security [142] utilized a molecularly imprinted (MIP) AgNPs composite for the LSPR sensing of an explosive taggant of trinitrotoluene (TNT), i.e., 3-nitrotoluene (3-NT). A one-step synthesis of MIP nanocomposite embedded with AgNPs was reported in the PEI matrix, which also serves as a reducing agent for Ag (I) to AgNPs. The synthesis steps are shown in Figure 12a, along with the sensing mechanism in Figure 12b. The sensor had a highly sensitive response due to the synergistic approach of LSPR and MIP, as shown in Figure 12c. LOD for 3-NT was reported to be 54.8 ng, with an extremely specific response in the presence of other NO_2_-containing molecules, as shown in Figure 12d. The approach can easily be implemented for portable readout sensors in remote detection and bomb-disposal robots. Another group [143] also reported porous silica embedded with silver clusters for the sensing of nitroaromatic and nitroaliphatic explosives. Thus, silver-based SPR and LSPR sensors are extensively reported in the literature for all possible sensing applications.

### 3.2. Surface-Enhanced Raman Spectroscopy (SERS)

**Figure 13:**
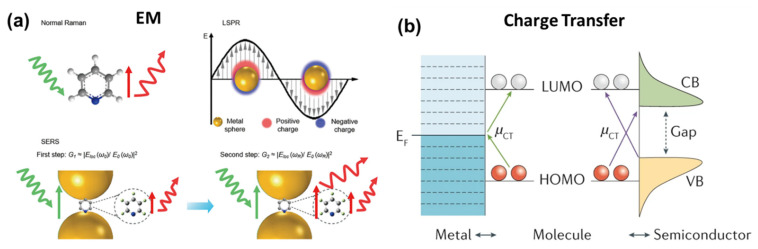
Schematic of enhancement mechanisms in SERS. (**a**) Electromagnetic (EM) enhancement mechanism in SERS, including the two-step enhancements illustrated in the nanogap of two metal nanoparticles. Reproduced with permission from [157]. Copyright 2018, American Chemical Society. Reproduced with permission from [158]. Copyright 2016, Springer Nature Limited. (**b**) Chemical enhancement through charge transfer (CT) between metal/semiconductor and the adsorbed molecule. The CT transitions (µCT) arrows show the CT directions. Red and white circles represent molecular orbitals. CB, conduction band; EF, Fermi level; HOMO, highest occupied molecular orbital; LUMO, lowest unoccupied molecular orbital; VB, valence band. Reproduced with permission from [159]. Copyright 2021, Springer Nature Limited.

As the name suggests, SERS is a technique to enhance the originally weaker Raman signal on the surface of plasmonic nanostructures. Before the discovery of SERS in 1974, [144], Raman was only used as a characterization tool within a limited area of interest. However, after the discovery of SERS, Raman spectroscopy gained tremendous attention in many avenues of research. SERS has the potential to detect chemical and biomolecules at a single-molecule level [15,145]. Therefore, is employed in various fields, e.g., medical diagnostics [15,17,146], food safety and security [147,148], environmental monitoring [149,150] and defense and security [151,152,153,154]. An appropriate laser excitation induces a local intense electromagnetic field in metal nanoparticles, which enormously enhances the Raman (as well as the fluorescence) signal of the molecule adsorbed on the surface. The magnitude of the signal enhancement is referred to as the enhancement factor (EF) [155], which is provided as

(2) $$EF=\frac{I\left(SERS\right)\times N\left(Bulk\right)}{N\left(SERS\right)\times I\left(Bulk\right)}$$

where *I(SERS)* and *I(Bulk)* are the signal intensities of the analyte molecule under SERS and the bulk (normal) Raman, respectively; *N(SERS)* and *N(Bulk)* are the number of analyte molecules being probed with SERS and the bulk Raman excitation, respectively. In general, the EF provided by equation 2 is in the order of 10^5^–10^6^; however, several reports claimed it to be up to 10^14^–10^15^ [145]. The SERS enhancement mechanism is still debatable, and, globally, physicists and chemists are continuously working to find more insights into this phenomenon. However, most of them agree on the two popular mechanisms known as (i) electromagnetic (EM) enhancement (plasmonic) and (ii) chemical enhancement (charge transfer) [20,21,156]. The first one has a higher contribution of about four folds, whereas the latter amplifies the signal by two orders of magnitude. Figure 13a shows the schematic of the enhancement mechanism (EM), and Figure 13b shows the charge transfer mechanism.

The quality of the SERS substrate is a prominent factor in the enhancement of the signal, which opens a new domain of research focusing on the fabrication and engineering of SERS-active substrates. Generally, there are two important criteria in developing a useful SERS substrate. First, the material should demonstrate superior optical responses (plasmonic activity) in the visible or NIR regime. Ag and Au qualify this very well, as the real and imaginary parts of their dielectric function have a large negative and a small positive value, respectively, in this region of wavelength. Second, the surface should be roughened or nanostructured to generate hotspots (the sharp curvatures or nanogaps on the structures) for sufficient E-field enhancement. For the highest enhancement, the molecules should be present in these hotspots. Here, anisotropy in nanostructures plays a very important role. Anisotropic nanoparticles, e.g., rods, flowers, stars, etc., provide higher surface area and a large density of hotspots as compared to isotropic particles such as nanospheres. The array, clusters or aggregates of these nanoparticles exhibit the enormous enhancement of the Raman signal [25]. The signal collected from the hotspot (also called hotspot SERS) is always at least 10^3^ times greater than the signal coming from other surfaces of the nanoparticles. For example, the dimer of two closely placed nanospheres only generates one hotspot region, whereas the dimer of nanostars or flowers generates many hotspots. Even if a single molecule is placed in the hotspot region, SERS will be observed. Therefore, single-molecule detection is possible and is attained using Ag hotspot SERS.

**Figure 14:**
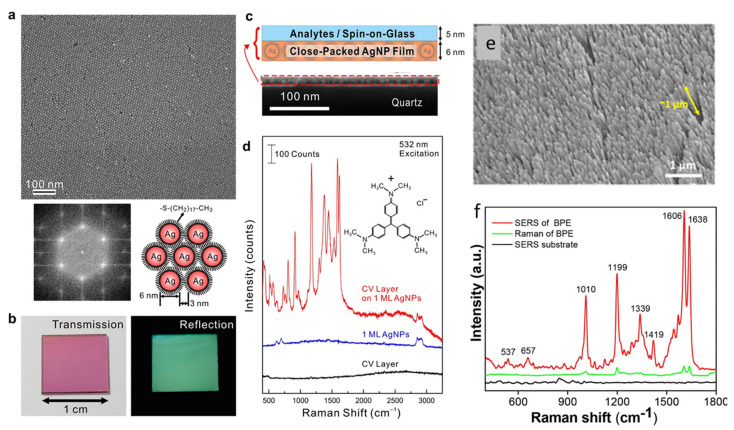
(**a**) FESEM image of a one-monolayer (1 ML), close-packed AgNP film on quartz, the diffraction pattern acquired from fast Fourier transform with an image area of 0.7 × 0.7 μm^2^ and the schematic showing the interparticle gap regulated by the thiolate chain length. (**b**) Transmission and reflection photographs illustrating the effect of collective plasmonic resonance. (**c**) Structure of the SERS substrate (close-packed AgNP film) and the analyte layer, which is a spin-coated layer of crystal violet (CV) molecules embedded in spin-on-glass (SOG); the cross-sectional SEM image shows the thicknesses of the CV/SOG layer and AgNP film are about 5 and 6 nm, respectively. (**d**) Raman spectra (vertically offset for clarity) obtained from a CV/SOG layer on AgNP film, a bare AgNP film and a bare CV layer. Reproduced with permission from [164]. Copyright 2015, American Chemical Society. (**e**) SEM of a GLAD-fabricated AgNR array. (**f**) Enhanced Raman signal of a BPE molecule deposited on the SERS-active array compared with normal the Raman of the molecule and substrate background signal. Reproduced with permission from [165]. Copyright 2020, American Chemical Society.

Not only plasmonic metals, such as Ag, Cu, Pt, Al and Cu, but many other nonmetallic materials (including metal oxides, sulfides, graphene, etc.) have also been investigated for SERS. However, the remarkable response of Ag is still unparalleled in this field. In addition, cost-effectiveness could be considered the second reason to select Ag over Au. In the following sections of this review, some important reports on silver-based SERS substrates and their applications will be discussed. Silver being the best choice amongst plasmonic materials, Ag-based SERS substrates have been fabricated by various methods and implemented in different domains of applications. The single-molecule detection limit by SERS has been attained by using Ag nanostructures [15]. AgNR arrays, vertically standing over a large-area substrate fabricated by GLAD, were optimized to provide a very high SERS enhancement. The effects of the aspect ratios of the NRs, as well as the gap between them and underneath reflecting silver thin film, were extensively studied experimentally and theoretically by Zhao’s group [160,161]. Subsequently, similar AgNR arrays were substantially explored in biosensing applications on the planar and flexible substrates by our group [162,163]. SERS-assisted single-molecule detection using a AgNP uniform monolayer was demonstrated by Chen et al. [164], as shown in Figure 14a–d. We reported an enhancement of about 10^8^ in the Raman signal of a probe molecule trans-1,2-bi-(4-pyridyl) ethylene (BPE) on a AgNR array fabricated over glass substrates, as illustrated in Figure 14e,f [165].

Further, to create a high density of hotspots (defined in the previous section), the substrates were modified into zigzag and multiple-armed geometry [96]. The effectiveness of the substrate is defined by the order of enhancement factor. Probe molecules with a high Raman cross-section are generally used, e.g., BPE, Rhodamine G (RhG), Methylene Blue (MB), Nitrothiophenol (NTP), Aminothiophenol (ATP), etc., for the determination of EF.

**Figure 15:**
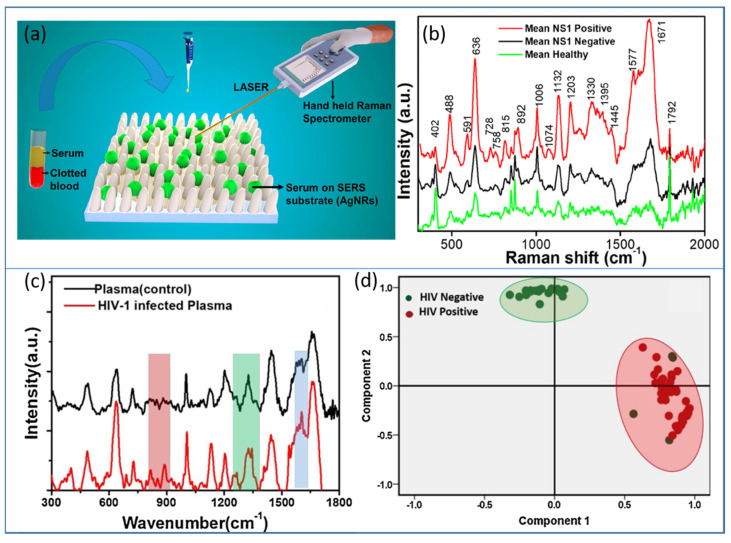
AgNRs as a portable SERS platform in disease diagnostics. (**a**) Schematic of the process of handheld SERS using a AgNR array. (**b**) The average SERS spectra of dengue-infected (NS1 positive), non-infected (NS1 negative) and healthy subjects. Reproduced with permission from [165]. Copyright 2020, American Chemical Society. (**c**) SERS spectra of healthy plasma (control) and HIV subtype D virus spiked in plasma (conc. 10^5^ copies/mL). (**d**) Principal component analysis (PCA) plot of HIV-1-positive and -negative plasma samples. Reproduced with permission from [166]. Copyright 2021, Elsevier.

Raman spectroscopy is considered a vibrational fingerprint of a molecule, and its advanced version, SERS, has overcome the low-intensity limitation of normal Raman. Therefore, SERS has become an established technique and has attracted immense attention for all the sensing applications in chemical and biosensing. There is hardly any domain left untouched by SERS-based detection. Recently, to make the technique user-friendly and cost-effective, various academic and industrial collaborations have developed portable/handheld or field-deployable SERS-based platforms, which have been implemented in biomedicine, defense and security. In view of rapid health monitoring for mass communities, especially in developing and highly populous countries such as India, SERS-based portable devices have been developed and tested on real clinical samples of dengue- [165] and HIV-infected [166] patients in hospitals. The label-free detection of the NS1 antigen present in dengue-infected patients was illustrated on an AgNR array, as illustrated in Figure 15a,b. The study was carried out over 100 subjects and the collected SERS data were successfully classified with the help of the principal component analysis (PCA) statistical tool [165]. The same strategy was applied for the detection of human immunodeficiency virus (HIV1) in clinical samples and also determined the tropism. The SERS spectra of control and HIV-infected blood plasma are shown in Figure 15c, and their classification using PCA is plotted in Figure 15d [166].

**Figure 16:**
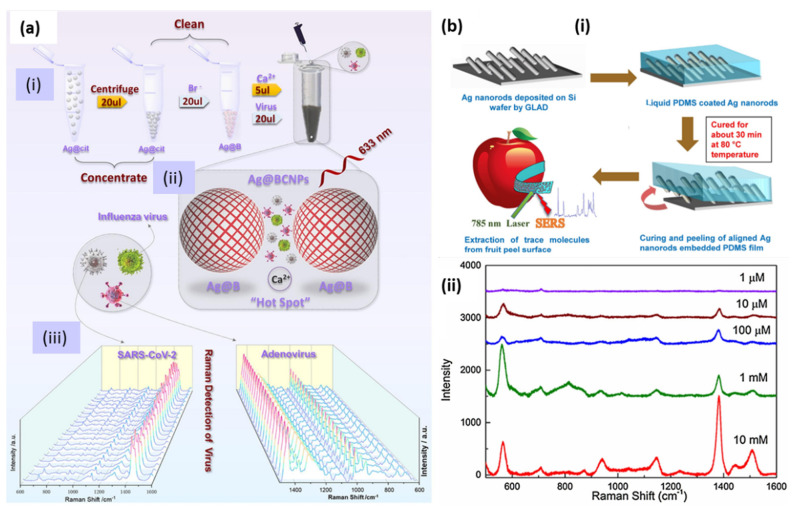
(**a**) (**i**) Schematic diagram of the preparation of silver-enhanced substrate and virus detection using SERS. Ag@cit: Silver nanoparticles obtained via the reduction of citrate; Ag@B: Silver nanoparticles modified by bromide ion; Ag@BCNPs: Ag@B with acetonitrile and calcium ions added. (**ii**) Conceptual schematic diagram of the relationship between the virus sample and the “hotspots” generated by the silver-enhanced substrate. (**iii**) SERS spectra obtained by 20 random groups of SARS-CoV-2 patients (10^4^ PUF/test) and HAdV (10^5^ copies/test) samples under the current method (Ag@BCNPs). Reproduced with permission from [167]. Copyright, 2022, Elsevier. (**b**) (**i**) Schematic showing the preparation of AgNR-embedded, PDMS-based flexible SERS substrate and (**ii**) SERS spectra of different concentrations of the pesticide thiram. Reproduced with permission from [148] Copyright, 2017, Elsevier.

Recently, the AgNP-based, label-free SERS detection of SARS-CoV-2, human adenovirus 3 and the H1N1 influenza virus were demonstrated. AgNPs were modified by utilizing calcium ions as aggregators, citrate ions were removed from the surface and acetonitrile was added to ensure the formation of high-quality hotspots. SERS measurements were performed on the samples obtained from 20 random groups of SARS-CoV-2 subjects. The schematic of the whole protocol is shown in Figure 16a [167].

Food safety is another domain in which SERS could be a promising tool. For detailed literature, readers are referred to the review papers [168,169]. As flexible substrates, AgNRs embedded in PDMS polymer were utilized as SERS tape for the detection of pesticide in fruits [148]. The flexibility and robustness of the substrate were examined under mechanical tensile strain conditions and using the scotch tape peeling test. A schematic of the substrate preparation is shown in Figure 16b(i) and the trace level detection of the pesticide thiram successfully achieved by the SERS spectra at different concentrations, as shown in Figure 16b(ii). Thus, SERS has proven to be a versatile tool for detecting and studying the kinetics of various chemical reactions. To obtain more insight into the present and future of this technique, readers are directed to several extensive reviews [158,170,171,172,173].

### 3.3. Surface Enhance Fluorescence Spectroscopy (SEFS)

Detection at the single-molecule level has gained considerable attention in the sensing and imaging community over the last few years. Among all the single-molecule optical spectroscopies, single-molecule fluorescence is the oldest and most widely applied spectroscopic technique due to inherited advantages such as noninvasive detection, fast and simple application and high contrast. Fluorescence is the property of certain organic molecules (fluorophores) to absorb and emit light through a transition in their electronic energy levels. However, for most fluorophores, fluorescence is limited by the low quantum yield, long relaxation time, poor photostability or photobleaching, which limit the single-molecule measurement due to a low signal-to-noise ratio. Purcell suggested in 1946 that the spontaneous emission properties of a molecule may be modified by controlling the external EM field in close vicinity [174]. Thus, the optical properties of a fluorophore may be effectively modified by keeping it in proximity to the nanostructured metal. The low fluorescence efficiency benefits from the interaction between fluorophores and the high nearfield enhancement caused by metal NPs, and it is referred to as metal-enhanced fluorescence (MEF) or surface-enhanced fluorescence (SEF) [67,175,176].

**Figure 17:**
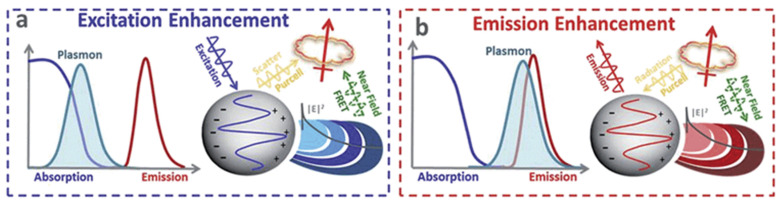
Depiction of MEF. (**a**) If the plasmon overlaps with the absorption of the fluorophore, an excitation enhancement is possible. (**b**) If the plasmon overlaps with the emission of the fluorophore, an emission enhancement is possible. Reproduced with permission from [16]. Copyright 2015, Royal Society of Chemistry.

MEF emerged as the most effective and important technique in improving the low fluorescence of molecules after the first classical interpretation of environmental effects on the excited state electronic transition of fluorophores by Drexhage in the 1970s [177]. Both the excitation and emission properties of a fluorophore may be modulated by controlling the EM field around it. Metal NPs can majorly enhance fluorescence efficiency with two processes [175]. Firstly, at the plasmon resonance wavelength, the nearfield around the NP is strongly enhanced, resulting in an increased absorption cross-section of vicinal fluorophores, which, consequently, enhances excitation and emission efficiency. Fluorescence is strongly quenched in the directly adsorbed molecules on the metal surface, but at a few nanometer distances, fluorescence can be strongly enhanced. The second process is the increased radiative decay rates of molecules due to coupling with metal NPs. Here, the excited state fluorophores may transfer their energy to surface plasmons, resulting in an increased decay rate and emission intensity from the metal–fluorophore complex system. This improves both the fluorescence intensity and photostability (less time in an excited state) of the molecule or fluorophore. In addition, MEF is confined to a volume surrounding the plasmonic particle and, hence, greatly limits the background signal from freely diffusing molecules. Thus, the interaction between a fluorophore and metal NPs, in general, may lead to the quenching of fluorescence or its enhancement depending on various parameters such as fluorophore–metal distance and relative orientation, shape and size of metal NPs; spectral overlap between LSPR modes; and fluorophores [178,179,180]. Chen et al. studied DNA linkers for the attachment of dye molecules to silver nanoprisms. They analyzed the near-filed effects leading to the enhancement in fluorescence intensity as a function of the spectral overlap between the LSPR resonance of silver nanoprisms and the dye’s emission and absorption spectra [181]. A detailed mechanism of MEF is available in a wealth of literature covering various possible mechanisms and factors responsible for the enhancement. A schematic depicting processes in MEF is shown in Figure 17 in terms of the spectral overlap of a fluorophore and a metal NP [16].

**Figure 18:**
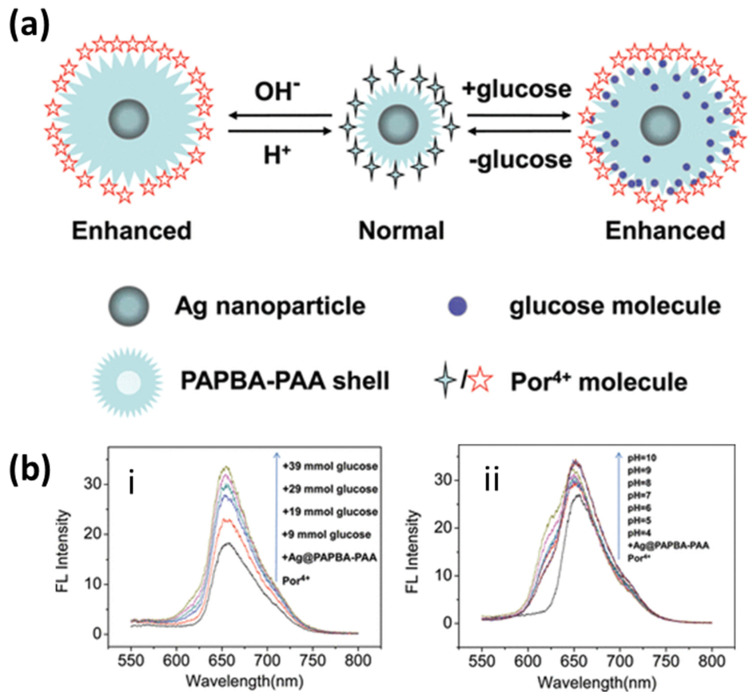
(**a**) Schematic illustration of the swelling–shrinking Ag@PAPBA-PAA hybrid nanoparticles with pH- and glucose concentration and the corresponding controllable MEF effects. (**b**) Fluorescence spectra of the Por4+/Ag@PAPBA-PAA nanocomposite as tuned by (**i**) various glucose concentrations and (**ii**) the pH of the environment. Reproduced with permission from [188]. Copyright 2012, American Chemical Society.

Numerous AgNPs based fluorescence enhancement methods have been reported in the literature with experimental and theoretical validations [182,183,184]. Several reviews highlight the critical applications of these methods for sensing [18,185,186]. Lin et al. proposed a silver nanoprism-based MEF sensor for the detection of sulfides in an aqueous solution. Atto550 has been used as a fluorophore attached via polymer COOH-PEG-SH and a streptavidin–biotin bond to silver nanoprisms. A series of nanoprisms were tested with resonance wavelength tuning in the range 500–900 nm. The highest enhancement in the fluorescence intensity was 10-fold, and it was obtained for prisms with an LSPR wavelength of 570 nm [187]. Ray et al. also demonstrated a several-hundred-fold fluorescence enhancement in Cy5 dye assembled on a silver NPs-dielectric-mirror (PDM) substrate. They elucidate the importance of single-molecule spectroscopy through these kinds of ensembles with a several-fold enhancement in fluorescence intensity and an up-to-10-fold enhancement in the decay rates of Cy5 [184]. Similarly, a core-shell silver-poly(3-acrylamidephenylboronic acid-co-acrylic acid) (Ag@PAPBA-PAA) structure was reported for pH and glucose sensing, incorporating porphyrin molecules (Por4+) as a fluorophore. The schematic of the fluorescence response and fluorescence spectra are shown in Figure 18. The response clearly indicates the distance-dependent fluorescence enhancement mechanism due to the swelling and shrinking of the PAPBA-PAA shell in response to the increased glucose concentration or pH [188].

**Figure 19:**
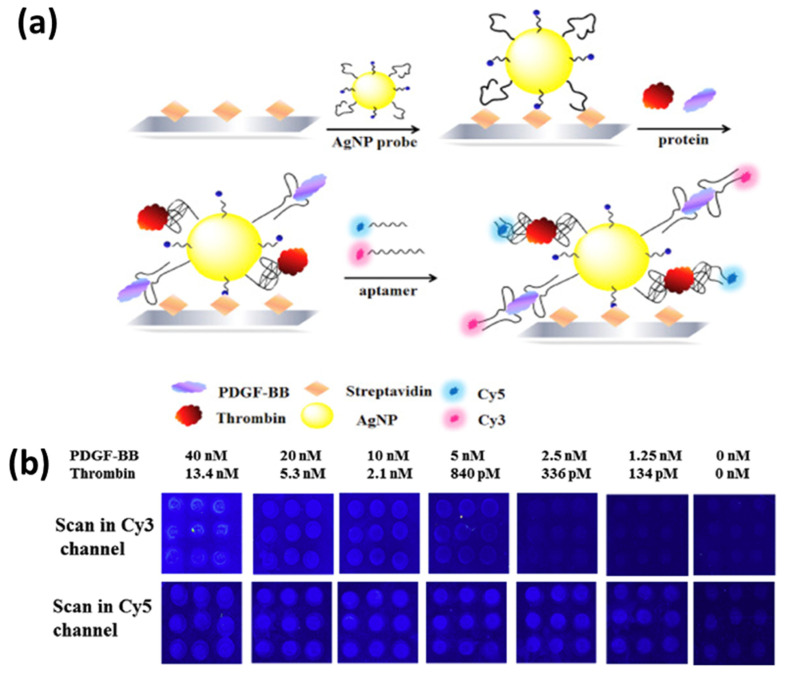
(**a**) Schematic for an aptamer-modified AgNP-based sandwich assay for multiplexed protein (thrombin and PDGF-BB) detection. (**b**) Fluorescence images for the multiplex analysis of the mixture of thrombin and PDGF-BB in different concentrations. Reproduced with permission from [189]. Copyright 2016, Elsevier.

Similarly, aptamer-modified AgNPs were used for the fluorescence-based detection of single and multiplexed proteins. The sandwich assay of aptamer-coated AgNPs as capture probes, as well as fluorophore-labeled aptamers as detection probes, improved the detection limit of thrombin and platelet-derived growth factor BB (PDGF-BB) by 80 or 8 times, reaching 21 pM and 625 pM, respectively. A schematic of the sandwich-type assay for multiplex detection is shown in Figure 19a, and the corresponding fluorescence readout for varying concentrations of thrombin and PDGF-BB is shown in Figure 19b [189].

As ordered metallic arrays have been found to be better substrates as compared to aggregated or randomly prepared nanoparticle suspensions, Ag nanorod arrays prepared with GLAD have also been explored for effective MEF substrates [190,191]. Enhancement factors have been analyzed in detail with a number of branches in zigzag Ag nanorod arrays. The detection limits of 0.01 pM have been achieved with an enhancement factor of 28 considering the hybridization of two oligonucleotides containing 33 base pairs using Alexa448. Here, the plasmon resonance of the nanorods may be tuned by varying their size to have a good spectral overlap with the fluorophore. Thus, it was concluded that a maximum 14-fold enhancement can be achieved for 7-fold zigzag nanorods due to an increase in the scattering intensity of the emission wavelength of the fluorophore and with an increase in the nanorods’ folding number. Several other sensors for the detection of insulin [192], heparin [193], proteins [194] and tetracycline [195] have been reported based on silver nanostructured MEF.

### 3.4. Surface-Enhanced Infrared Absorption (SEIRA)

Infrared (IR) spectroscopy measures the vibration of molecular bonds by considering the absorption in the mid-IR spectral region (3000–600 cm^−1^). IR spectra are intrinsically endowed with the chemical fingerprint of the molecule and, hence, may be leveraged for the direct measurement of molecular mechanisms. However, conventional IR spectroscopy has limitations such as low sensitivity and difficulty measuring aqueous solutions due to the IR activity of water. The enhancement of IR absorption in the molecules adsorbed on metallic nanoparticle surfaces is referred to as surface-enhanced infrared absorption spectroscopy (SEIRAS) and can significantly overcome the limitations of IR spectroscopy. The first study on SEIRA was reported after almost a decade of SERS by Hartstein et al. [196]. The mechanism of enhancement is quite similar to the SERS phenomenon, as Raman and IR are just two complementary vibrational spectroscopies. The detailed mechanism can be found in a number of review articles, chapters and books dedicated to this topic [19,197,198,199]. Of note, the localized EM field around metal NPs and the charge transfer between the molecule physiosorbed or chemisorbed on the surface of metal NPs give rise to a 10–1000-fold enhancement of IR absorption as compared to conventional techniques. Although the enhancement factors are much lower than SERS, the much higher infrared absorption cross-section compared to Raman scattering has maintained the interest of researchers in this field. Depending on the polarization and angle of incidence of the IR light, enhanced spectra may be collected in the attenuated total reflection mode (ATR), the transmission mode or the diffuse reflection mode.

The potential of SEIRA has been recently explored over citrate-stabilized AgNP substrates for the identification of microorganisms: *Candida albicans* (*C. albicans*), Escherichia coli (*E. coli*) and Staphylococcus aureus (*S. aureus*). It was concluded that SEIRA provides more explicit molecular information about these species as compared to conventional FTIR spectroscopy [200]. Similarly, a novel chalcogenide waveguide sensor using Ag-island film was reported exploiting SEIRA for the detection of the gaseous and liquid phases. Various thicknesses of Ag-island film were fabricated for the best sensing performance, and it was found that a 1.8 nm thickness provides the best results. The absorbance enhancement factors of >1.5 for ethanol (C_2_H_6_O) at 1654 nm and >2.3 for methane gas (CH_4_) at 3291 nm were also obtained. Lift-off and GLAD were used for the fabrication of this SEIRA waveguide sensor [201]. The application of this sensor for shale gas measurement was reported, which is very useful for exploring natural resources. Similarly, an ATR SEIRA analysis of fatty acids was also successfully performed on silver nanoparticles [202]. Apart from this, many recent review articles have covered the field of biosensors using SEIRA [203,204]. The aforementioned plasmonic sensing techniques and their applications are summarized in Table 1.

## 4. Conclusions and Outlook

In the current scenario, most of the research on nanotechnology has somehow moved toward the involvement of plasmonics due to the attraction capability of plasmonic nanomaterials for light confinement and manipulation. In other words, plasmonic metal nanostructures act as optical antennae to covert light into localized electric fields and route it to a desired location with nanometric precision. This field is growing so profoundly that the entire fraternity of nanoscience is engaged in understanding more insights into the phenomenon and also in developing new technologies employing this extraordinary feature. In this review article, we exclusively discussed the most competent candidate, Ag, among all plasmonic materials. Although Au is an equally accepted metal, we have emphasized the choice of Ag over Au. The outstanding plasmonic response in the desired spectral range and the cost-effectiveness have overpowered the issues of biocompatibility and the stability of Ag nanoparticles. Different facile synthesis methods have also played a vital role in making it more accessible. Here, major techniques for AgNP chemical synthesis and physical deposition have been discussed and reviewed to date. Recent developments in the field of controlled synthesis and the assembly of Ag nanostructures involving nanoskiving and DNA origami are still motivating researchers to unravel the new dimensions of plasmonics and its applications.

The localized plasmonic electric field resonating with external light has enriched spectroscopic techniques, e.g., Raman, fluorescence and UV-vis. Therefore, metal nanoparticles enable surface-enhanced spectroscopy such as SERS, SEF and SEIRS, which are emerging as techniques for the sensing and detection of chemicals and biomolecules. They have been found to have promising applications in numerous fields. In this article, we tried to cover the applications of silver nanostructured substrates in the above techniques with major emphasis on SPR and SERS-based plasmonic applications, e.g., biosensing, environmental monitoring, security and food safety. It is now well understood that the limits and commercial applications of plasmonic sensors can be pushed with an in-depth understanding of the underlying theoretical principles of each of these techniques. Each technique has its own limitations and advantages, which must be carefully taken into account to develop the final prototype for a particular application. The commercialization of SPR-based sensors is promoted due to their high sensitivity and the miniaturized devices facilitated by optical fiber substrates. However, the realization is still limited by their reusability and durability due to the requirement of designing the specific sensing layer. Fluorescence-enhancement mechanism caused by metals provides a boost to the well-established field of fluorescence and FRET-based sensing, improving their sensitivity and providing low-cost, easy-to-handle, readily available commercial systems. However, the low quantum yield of visible fluorophores and tuning the metal NP–fluorophore distance in such systems remain challenges to be addressed in order to carefully avoid fluorescence quenching and to realize enhancement. Similarly, SEIRA sensors have gained advantages from the enhancement of IR signals by metals up to the order of 10^4^, but they are still limited by the inherited properties of IR spectra, such as interference from aqueous solutions due to the strong IR signal from water. Among all the surface-enhanced spectroscopies, SERS has proven to be the most reliable and sensitive technique, as it can provide the direct fingerprint of target chemical species, avoiding any interference possibilities. This has enormously guided researchers all over the globe to explore SERS-based sensors for a plethora of applications. As Raman spectrometers are generally large and expensive, this has limited the point-of-care devices based on SERS as compared to the compact instrumentation required for SPR and SEF studies. Thus, now the research is focused on miniaturizing SERS-based systems by developing paper-based substrates, microfluidic platforms and palm-sized spectrometers to commercialize SERS as a promising detection tool.

Thus, plasmonics has emerged as an important fundamental science for resourceful technologies. Apart from sensing, many new applications for metal NPs are rapidly emerging in the fields of plasmonic catalysis, circuitry and quantum computing. The plasmon decay in metals generates highly energetic electrons (hot electrons) and localized heating, which can be utilized in many ways, such as modulating various chemical reactions and the conversion of solar energy to chemical energy. Water splitting, hydrogen, oxygen generation, CO_2_ reduction and many more make up the attraction to plasmonics nowadays. The fast-paced growth of plasmonics is evidence of its versatility in next-generation technological solutions for society, and it still inspires researchers worldwide with open questions.

## Figures and Tables

**Table 1:** Ag-based plasmonic sensing techniques and their applications for sensing various analytes.

Sensing Technique	Ag Substrate Structure	Sensing Method	Sensing Layer	Analyte	Ref.
SPR	Thin film	Thermal evaporation	ZnO/CNT nanocomposite	Catechol	[118]
SPR	Thin film	Thermal evaporation	SnO_2_ thin film	Ammonia gas	[86]
LSPR	Nanoparticles	Chemical reduction	PAH capped Ag NPs	Hydrogen peroxide	[123]
SPR/LSPR	Thin film and NPs	Thermal evaporation and chemical	Cholesterol oxidase in polyacrylamide gel and graphene oxide	Cholesterol	[128]
SPR	Thin film	Thermal evaporation	Anti-NS1 antibody over SAM layer	Dengue	[129]
LSPR	Nanotriangle array	NSL and e-beam evaporation	ACE2 protein	SARS-CoV-2	[130]
SPR	Thin film	Thermal evaporation	MIP	Erythromysin	[140]
LSPR	AgNPs	Chemical reduction	MIP	3-NT	[142]
SERS	AgNP film	Chemical	Closely packed AgNP film on quartz	CV	[164]
SERS	AgNPs	Chemical	AgNPs modified by bromide ions, acetonitrile and calcium ions	SARS-CoV-2, H1N1 influenza virus, Human adenovirus-3	[167]
SERS	AgNR array	GLAD	AgNR array embedded in PDMS	Thiram	[148]
SERS	AgNR array	GLAD	AgNR array on glass	Dengue	[165]
SERS	AgNR array	GLAD	AgNR array on glass	HIV-1	[166]
SERS	Zigzag Ag–Al array	GLAD	Ag–Al zigzag array on glass	BPE	[96]
SERS	AgNR bundles	NSL and electrodeposition	Porous AAO coated with AgNR bundles	Phenolic pollutants	[75]
SERS	Ag nanotriangles	Chemical reduction	Ag nanotriangles on silicon	Picric acid and ammonium nitrate	[153]
SEF	Ag nanoprisms	Chemical reduction	ATTO550 attached via COOH-PEG-SH and streptavidin–biotin	Sulfides	[187]
SEF	AgNPs	Sputtering	AgNPs on silica	Cy5	[184]
SEF	AgNPs	Chemical	Ag@PAPBA-PAA gel embedded with porphyrin as a fluorophore	pH and glucose	[188]
SEF	AgNPs	Chemical	Aptamer-modified AgNPs	Thrombin and PDGF-BB	[189]
SEF	AgNR array	GLAD	Biotin-coated AgNRs	Neutravidin and DNA	[190]
SEIRA	AgNP suspension	Chemical	AgNP direct detection	*C. albicans*, *E. coli*, and *S. aureus*	[200]
SEIRA	Ag nanoislands film	GLAD	Direct detection by Ag nanoislands	Ethanol and methane gas	[201]
SEIRA	AgNPs	Chemical	Direct detection	Fatty acids	[202]

## Data Availability

Not applicable.
